# Application of botulinum toxin in maxillofacial field: Part III. Ancillary treatment for maxillofacial surgery and summary

**DOI:** 10.1186/s40902-019-0226-0

**Published:** 2019-10-24

**Authors:** Kyung-Hwan Kwon, Kyung Su Shin, Sung Hee Yeon, Dae Gun Kwon

**Affiliations:** Department of Oral and Maxillofacial Surgery, College of Dentistry, Wonkwang University, Iksan, South Korea

**Keywords:** Botulinum toxin, Clinical application, Maxillofacial field, Dental implant, Orthognathics, Orthodontics, Maxillofacial pain

## Abstract

Botulinum toxin (BTX) has various therapeutic indications: bruxism, square jaw, facial wrinkle, oral ulcer and maxillofacial pain, etc. In this paper, we will discuss the effectiveness of using BTX in dental implant surgery and orthognathic and orthodontic treatment. We summarized the clinical application of botulinum toxin in the maxillofacial field at the finale.

## Background

Botulinum toxin (BTX) weakens the muscle and the temporary muscle paralysis may improve post-operative recovery and healing [1]. In the case of multiple implants or immediate loaded implants, osseointegration can be delayed by excessive functional forces in patients with a parafunctional habit [2]. The muscular relaxation by using BTX can be beneficial by allowing implant structures better osseointegrated [3]. In maxillofacial surgery, BTX was useful in the wound healing process of facial lacerations requiring surgery. And also, BTX has been used to reduce the displacing forces on the fracture site [4]. By reducing the force of masseteric muscle, orthognathic and orthodontic treatment may be improved. However, the research about the clinical use of botulinum toxin for implant and maxillofacial surgery is yet insufficient. It may be necessary to discuss about application of botulinum toxin in these fields.

## Main text

### Clinical application of botulinum toxin in immediate loading and immediate postextraction implant surgery

#### Implant occlusion and osseointegration

Basically, an implant is connected by a screw structure, and when lateral pressure is applied, it will exert a torque causing loosening of the prosthesis fixing screw of the upper structure or loosening of the abutment screw, which ultimately leads to the disintegration of the implant fixture. Additionally, according to the study of Richter, even though the effect of excessive occlusal pressure acting on natural teeth and implants do not differ each other much, the grinding and clenching of natural teeth, such as bruxism, cause excessive attrition of teeth, and for the case of implants, it results in damage to the implant fixture or the upper structure of implants [5]. Isidor noted that most of the implants that received excessive occlusal pressure are having osseointegration failure and would require measures against non-functional or parafunction mostly caused during the sleep [6].

#### Countermeasure for bruxism

A study by Duyck et al. have also shown that if the number of implants decreases, the amount of bite pressure exerting on implants increases [7]. Therefore, increasing the number of implants withstanding the bite pressure is considered one of the measures against parafunction. However, due to financial issues and the location of implants, there is a case where only a small number of implants are used for prosthetic restoration. According to the report by Maan et al., the muscle activities of the masseter muscles undergone occlusion therapy such as bite plane treatment have reduced [8]. The study of al-Quran et al. has reported that occlusion therapy has ability in reducing the force exerted to the implant by inhibiting the activity of the masseter muscles [9]. The use of botulinum toxin, which is known to reduce bite pressure by 20–40% started upon the above theoretical base. It is worth considering botulinum toxin treatment as a way to increase the osseointegration of implants and to reduce the level of bite force exerted on the implant by injecting botulinum toxin into the muscles forming bite forces such as the temporal muscle, masseter muscle, and medial pterygoid muscle.

According to To et al., masseteric muscle mass was reduced by 31% on ultrasonic and electromyogram 3 months after the injection. They reported that out of 9 masseter muscles used in the test, 6 maintained atrophic states for 1 year [10]. Therefore, we can assume that reduced biting force due to atrophy of masseter muscles aid in the prevention of bruxism by temporarily inducing an environmental change in the occlusion. Generally, biting force is known to decrease up to 20–40%; because muscle relaxation more effectively progresses at resting, it is known to reduce excessive clenching of teeth or bruxism phenomenon during sleep.

Alberktson suggested six important factors affecting the osseointegration of an implant: (1) bioavailability of implant material, (2) design of the implant, (3) surface of the implant, (4) condition of the bone, (5) surgical technique, and (6) condition of the load. Among these, the condition of the load can have a profound impact on osseointegration in patients with a parafunctional habit. In the patient with a parafunctional habit, the reduction of occlusion force will provide the patient with time to help osseointegration [11]. Therefore, we can infer from the information that botulinum toxin may be used as an adjunct therapy to increase the success rate of immediate loading and immediate postextraction implantation in case the patient has a parafunctional habit or strong bite force by reducing the occlusion force resulting from masticatory muscles.

#### Immediate loading implantation and immediate postextraction implantation

The success of treatment in immediate loading implant and in the immediate postextraction loading implant largely depends on various factors. Factors known to affect the success of immediate loading implants are divided into four categories: surgery-, host-, implant-, and occlusion-related factors. Surgery factors include primary implant stability and surgical technique. Host factors include the amount and quality of cortical and trabecular bone, wound healing, modeling/remodeling activity, etc. Implant factors include design, surface texture, and dimension. Along with botulinum toxin, the topic of our interest, the occlusal factors include level and quality of occlusal force and design of the prosthesis.

Adjusting the functional force is one of the factors that help to successfully complete treatment of immediate loading implant. Sagara et al. observed a more crestal bone loss in a loaded 1-stage implant compared to a 2-stage implant [12]. This is because early loading disrupts the osteogenic ability of bone that could replace bone necrosis caused by surgical trauma, resulting in bone absorption at the time of early occlusal loading during wound healing [11]. Vertical force exerted during the function is less harmful than oblique or horizontal force in terms of stability of an implant. Thus, bruxism or occlusal overload should be contraindicated in the immediate loading implant because of high failure rates [13]. However, Ganeles et al. reported that only one case had failed due to bruxism among 161 immediate loading implants [14]. Unfortunately, up to date, there is insufficient scientific information related to parafunctional habit in the immediate loading failure. Columina et al. reported a success rate of 97% of immediate loading implant; however, the failed implants (2 out of 61) were due to occlusal pathology and intraoral muscle tension [15].

**Fig. 1:**
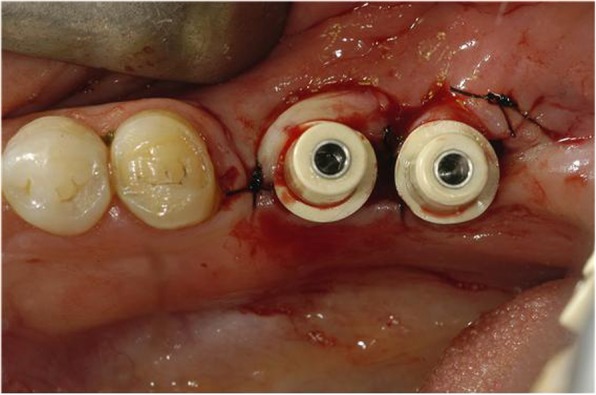
A patient planned with placement of immediate loading implant in #36 and #37. Planning to place Pitt-Easy Bio-Oss® implant, perform resin restoration using A.G.T® post

**Fig. 2:**
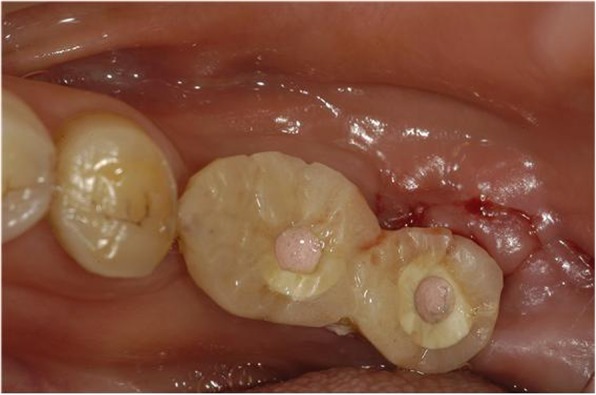
#36 and #37 restored with resin, 1 week after implant placement. BTX-A injection was performed on the masseter muscle a week before implant placement

**Fig. 3:**
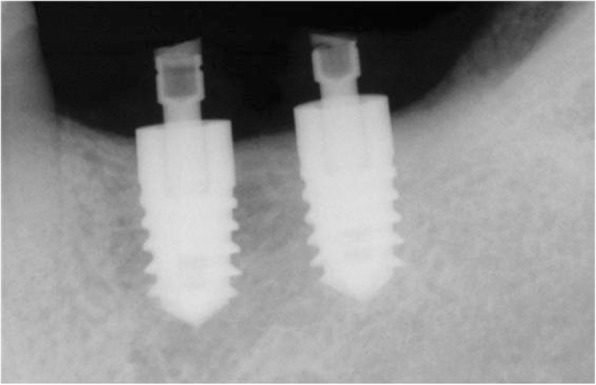
#36 and #37 Immediate loading was performed along with implant placement. Since the occlusion force was reduced by 30%, there was no problem with the fracture of the resin restoration or the initial fixation of the implant. It is also advised to use botulinum toxin as an adjunct

In the case of such immediate loading implants, the use of botulinum toxin can be considered a method of controlling the potential occlusion load. Although in clinical settings we are aware of various factors that act on success rate while implanting the implants, we often overlook the factors of the occlusal load. From this standpoint, we can expect that botulinum toxin will play a role in raising the success rate by reducing the occlusion load by about 30% through its use (Figs. 1, 2, and 3).

#### Selection of injecting area

There are several ways to reduce the maximum occlusal force in the case of immediate load implant placement. The first method is to adjust the occlusion by reducing the interference or the lateral force of immediate loading, and the second is to achieve a functional occlusal force that an articulating paper can be easily removed during the maximum occlusion. In addition to that, you can use botulinum toxin. Among the four masticatory muscles, the masseter muscle and temporal muscle are known to contribute to the largest occlusion force during the mouth closure. We recommend injecting 25 BU (a dose of BTX-A or Botox, the dose corresponds to 100 DU-Dysport) into each of the masseter and temporal muscles, bilaterally. The occlusal force itself is reported to be reduced from 20 up to 40%. You can use the same injecting method as in the botulinum toxin treatment of masseter muscle hypertrophy patients or bruxism patients.

**Fig. 4:**
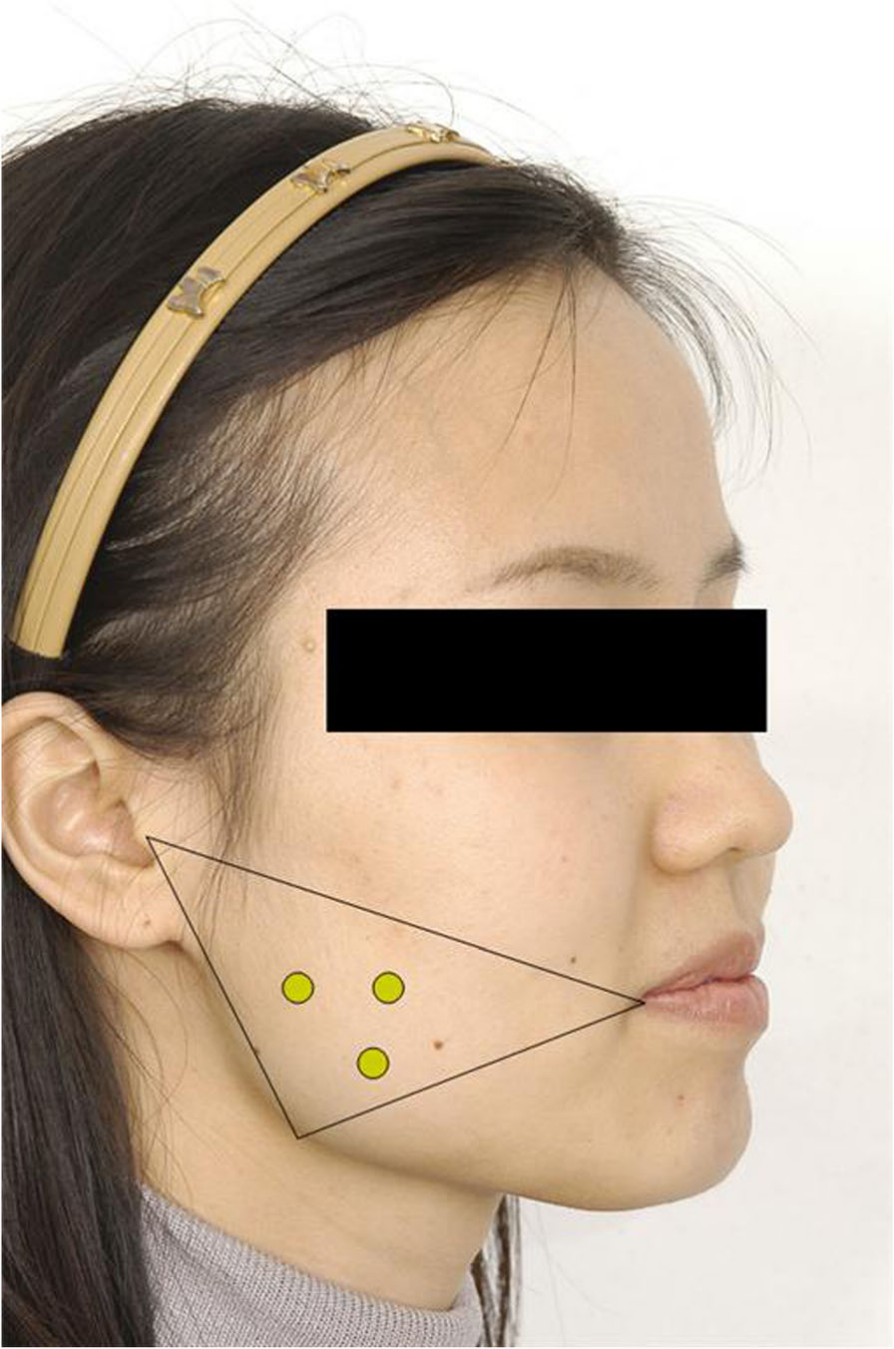
Picture demonstrating the three injection points. Injection point is within the triangle made by a line connecting the mouth corner, the tragus, and the angle of the mandible. Inject deep into the muscle, careful not to touch the periosteum

The dose of botulinum toxin injected into the masseter muscle is generally about 25 BU, and in the case of unilateral masseter muscle hypertrophy or varying occlusal force, we do not have to change the number of injections or reduce the dose. As we mentioned earlier, the injection point is located within the triangle made by the lines connecting the corner of the mouth, tragus, and angle of the mandible. Inject into the thickest area on the muscles during mouth closure. The number of injections may vary depending on the surgeon, but we recommended injecting 3 or 4 points where the distance between each injection points do not exceed 1 cm (Fig. 4).

### Clinical application of botulinum toxin in orthognathic and orthodontic patients

Orthodontic treatment is a method that helps to restore a normal tooth arrangement and occlusion. Nowadays, people prefer orthodontic treatment to achieve aesthetical and functional improvement of the face. Orthodontic treatment is seeking to improve soft tissue by changing bone tissue adjacent to the teeth. Under this paradigm, the esthetic importance of soft tissue and the desire for improvement are more and more increasing. When an asymmetry of the face is corrected by orthognathic surgery on skeletal structure, the soft tissue improvement occurs after a period of at least 6 months to a maximum of one and a half years. We have been studying the use of botulinum toxin in accelerating the transformation and balancing out asymmetry in the volume of muscles. In orthodontics, the use of botulinum toxin can be summarized into four ways.

Firstly, by reducing occlusion force, it can be used to prevent delaying of tooth movement caused by occlusal force.

Secondly, by reducing muscle activity, it can be used to improve the asymmetric volume of soft tissues due to the asymmetrical face. Thirdly, it can be used to reduce the soft tissue in the asymmetrically activated part of the masseter muscle by reducing the volume of the muscles. Lastly, scar formation could be minimized due to inhibition of the contraction of the scar tissue.

It can be used to reduce the asymmetrical volume of soft tissues after orthognathic surgery. Patients with skeletal malocclusion are often characterized by asymmetric growth patterns. Along with Class III malocclusion, there can be asymmetric growth patterns in both soft tissues and skeletal structures. There may be various causes; however, when skeletal malocclusion is corrected, the improvement of the asymmetrical pattern of soft tissue will take at least 6 months up to one and a half years.

**Fig. 5:**
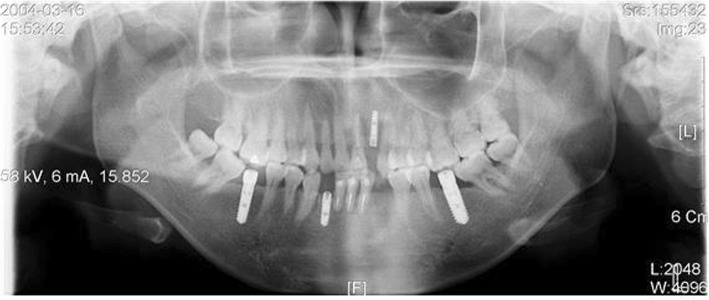
A case in which the stability of the implant is being threatened due to stimulation on gingival former of the implant which resulted by mesial tilting of #47 and #37 after implant placement. Extraction of #38 and #48 following implant placement and uprighting #37 and #47 with SAS was attempted, which was not easy due to the patient’s occlusal force. Use of botulinum toxin is considered in such a case

**Fig. 6:**
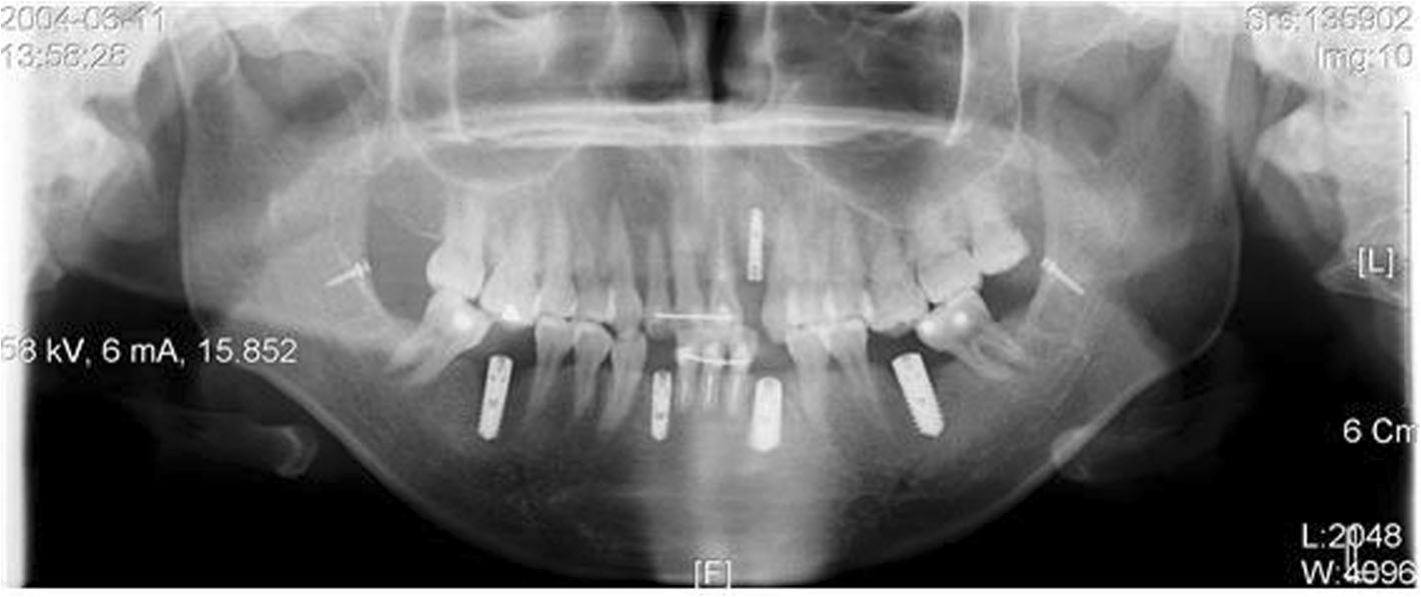
In 3 months after injecting botulinum toxin into both sides of the masseter muscle and using SAS to upright #37 and # 47, teeth repositioned to normal, and botulism toxin lowered the occlusal force acting on the implant

Besides, botulinum toxin can be used to reduce muscle activity in a patient with gummy smile, and it can also be used to significantly reduce the time needed to restore the tooth axis by reducing occlusal force during uprighting the molars (Figs. 5 and 6).

Botulinum toxins can be used along with orthognathic surgery, and also in conjunction with various procedures for shortening the period of orthodontic treatment. There are some challenges and risks in expanding the scope of application of botulinum toxin; however, since it has fewer side effects and is relatively safe, it is worth trying. More verification should be made on muscle contraction and muscle activity, and various approaches should be made for the head and neck area.

So far, we have discussed various uses of botulinum toxin in the maxillofacial field. In the near future, we hope that there will be an opportunity to discuss in detail and study the application of botulinum toxin in various aspects of the maxillofacial field.

### Summary: clinical application of botulinum toxin in maxillofacial field

Botulinum toxin type A, which is a polypeptide neurotoxin derived from anaerobic bacterium clostridium botulinum, has been used reliably for the treatment of strabismus, blepharospasm and palmar hyperhidrosis, and others and has recently been widely used for the purpose of improving facial wrinkles. Particularly in the maxillofacial surgery area, aside from wrinkle improvement, it has a wide scope of application, such as square jaw and facial asymmetry due to facial nerve abnormalities, and the treatment effect is quite satisfactory. In anticipation of the large use by maxillofacial surgeons and even dentists, this paper aims to learn about the historical background, pharmacological mechanism, possible complications, applicability in the maxillofacial area, etc. of the botulinum toxin use.

#### Historical background

Botulinum toxin was first discovered in a decayed sausage in 1829, making it being called as a sausage poison, and is known to have an inhibitory effect on stimulation. Justinus Kerner, a doctor and author, mentioned the possibility of medical use of this toxin for muscle relaxation if there is a disorder in overstimulated motor system [16]. In 1897, Belgian microbiologist Evan Ermengen succeeded in separating pathogens from the bowels of a patient who ate a decayed sausage, and named this as *Bacillus botulinus* [17]. *Botulus* is a Latin expression of sausages. Schantz succeeded in mass-producing large quantities of toxin [18]. It would be said that the basis for the clinical application of this toxin was laid when Burgen discovered that botulinum toxin works by presynaptic acetylcholine inhibition in 1949 [19]. Botulinum toxin A (BTX-A) was first used in the treatment of strabismus by Alan Scott in 1973, and the US Food and Drug Administration approved the use of this toxin for experimental treatment of strabismus in 1979 [20].

The use of this toxin was expanded to blepharospasm in 1958, and other groups started to pay attention to the use of this toxin. In 1982, Jean Carruthers, an ophthalmology professor at the University of Vancouver in Canada, participated in a clinical trial of the strabismus, discovered by chance that the wrinkles in glabella region (middle of the forehead) decreased during the BTX-A treatment in a patient with blepharospasm, and observed that the skin maintained its firmness for a few months, confirming that this is due to relaxation of facial muscles. This subsequently began to be used for the treatment of wrinkles in the eye, nose, and jaw [21–23]. The clinical success of this toxin has led to a wide use in the USA and has been achieving good results by being combined with anti-wrinkle treatment in dermatology, otolaryngology, ophthalmology, ophthalmology, plastic surgery, and maxillofacial surgery.

#### Pharmacological mechanism of BTX-A toxin

Clostridium is an anaerobic bacterium that produces seven serotypes of toxin, or botulinum toxins A-G. BTX-A is used for therapeutic purposes and BTX-B (neuroblock) is used for some neurological indications. BTX-F is currently under clinical trials and BTX-C is currently only being tested [24, 25]. BTX-A has a molecular weight of 150 kDa and is consisted of two subunits with a single disulfide bond. Therefore, the toxin is relatively unstable and is easily deactivated when subjected to mechanical or heat stimulation. The 147-kDa heavy chain has strong affinity (irreversible) for the sialogly-coprotein-specific receptor located in the plasma membrane of cholinergic nerve ending. This induces a receptor-mediated endocytosis. Light chain (52 kDa, Zn^2+^ protease), which is responsible for toxicity, splits inside the cell and deactivates the synapse-specific protein. This deactivation prevents the fusion between a vesicle filled with acetylcholine and a plasma membrane [26].

Neuromuscular block causes atonal paralysis in skeletal muscle and atonia of smooth muscle in parasympathetic nerve endings, causing dysfunction of organs controlled by parasympathetic nerve, hypohidrosis, and anhidrosis.

After intramuscular injection of the toxin, the first clinical effect after appears 24–72 h later and shows the best effect after 1–2 weeks. The effect typically lasts 3–6 months and can last up to 7–9 months if it is injected again [26]. During this period, the initial function of the nerve endings is restored through the decomposition of the toxin by proteolysis and the creation of a new SNAP-25 (synaptosome-associated protein). In addition, regeneration can occur through spouting of the nerve ending and the creation of new synapsis. The changes in the muscle with atrophy were also demonstrated by animal experimentation, which was fully recovered 4–6 months later. In humans, a sustained atrophy was not observed after repeated injections.

The capacity is determined by the activity, rather than the molecular weight. While the biological activity is expressed as mouse unit, 1 MU refers to the 50% lethal dose (LD50) when injected in the abdominal cavity. The FDA classified BTX-A as a safe drug for treatment because the amount of BTX-A used for disease treatment purposes is diluted to 25- or 100-fold than LD50. This toxin neither passes through neither the blood-brain barrier nor penetrates the skin. Furthermore, it is reported that the toxin’s capacity and the duration of action are not related with the dose, but this is not certain.

**Fig. 7:**
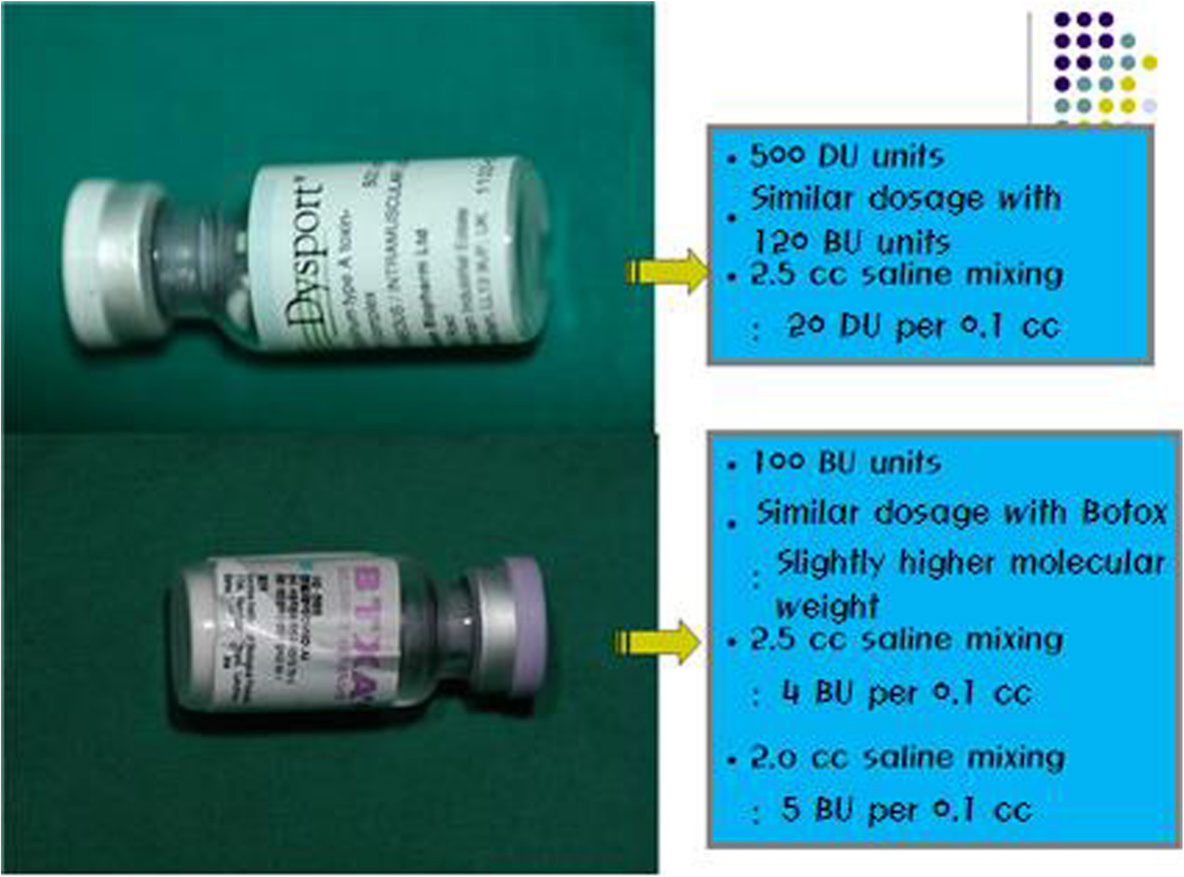
Brand names for botulinum toxin A include Dysport, BTX-A, Botox, etc. The drugs differ from each other in their unit and possess different methods of injection depending on their method of dilution. For the treatment of bruxism, in the case of BTX-A

Currently, BTX-A is mainly commercialized and supplied by two companies (some are being developed and commercialized in China but its use is not very popular. One is Dysport, produced by the Ipsen company in Wrexham, England, and the other is Botox, produced by an Allergan company in Canada and the USA (Fig. 7).

The potency of each unit of the two products is about three times different. In other words, Allergan is three times more potent. Therefore, 1 U of Botox corresponds to 3-5 U of Dysport. Among the differences between the two products, the characteristic difference is that Botox needs to be stored in freezer, while Dysport can be stored in refrigerator and has a longer storage period than Botox, making no big difference in the effect when it is stored in solution for about a month by many surgeons (Table 1).

**Table 1:** Comparison of Botox and Dysport

	Botox	Dysport
Components	Clostridium botulinum toxin	Clostridium botulinum toxin
Vial	Type A 100 U	Type A 500 U
Effects	1 Botox unit	3~4 Dysport unit
Half-life	Dry: 24 months Solution: 5 h	Dry: 1 year Solution: 8 h
Manufacturer	Allergan, Irvine, CA, USA	Ipsen, Ltd., Wrexham, UK
Dilution	100 Unit + 2.5 ml NaCl Physiologic solution : 2 U in 0.05 ml : 4 U in 0.1 ml	500 Unit + 2.5 ml NaCl Physiologic solution : 10 U in 0.05 ml : 20 U in 0.1 ml

#### Migration factors

The concern in using a toxin is the risk of toxin migration. In other words, it can move from the injection site to the unwanted site and cause complications. In particular, the following three areas have a large risk of migration. When injecting toxin to corrugator muscle above the eyebrow, blepharoptosis can occur when toxin migrates downward. The migration distance between the injection site and levator palpebrae muscle is 2 cm. When injecting toxin to the outer side of orbicularis oculi muscle, toxin can migrate to levator palpebrae muscle. The migration distance is 2 cm as well. When injecting to frontal muscle, toxin can migrate downward of frontal muscle, causing eyebrow to sag downward. The migration of the toxin is affected by amount of injection, direction of injection, and bleeding on injection [27].

1. *Amount of injection*. The possibility of migration can be reduced by injecting a small amount of highly concentrated toxin for the area where complications are concerned during migration. For the areas that do not concern on migration, even spread to the desired area can be expected by injecting a low concentration of toxin (the total amount is constant but the volume is large).
2. *Direction of injection*. While most injection uses an insulin injection syringe, the needle bevel needs to face the intended area. For example, when injecting to the corrugator muscle, the beveled part of the needle needs to face the opposite side of the levator palpebrae muscle.
3. *Bleeding on injection*. There may be fine bleeding upon injection occasionally, and this can cause ecchymosis in some cases. As this moves downward due to gravity, it can migrate the toxin. In such cases, it is possible to prevent this to some extent by pressing down the bleeding area with a finger immediately.

#### Immunity

The neutralizing antibodies can be the cause of treatment failure, and this often occurs when a large quantity of BTX-A is repeatedly injected (secondary treatment failure). This deactivated antibody results in partial or complete loss of the effect. To prevent this immune resistance, it is necessary to use as small but effective quantities as possible. The interval between injections should be more than 8–12 weeks. The literature reports antibody production in 3–10% of toxin [28].

#### Patient preparation

As same with other cosmetic procedures, check neural and hemodynamic diseases, medication allergy, etc. The injection should be conducted in a semi-seated or seated position, and the injection site should be cleaned with a non-alcoholic disinfectant before injection.

#### Preparation of toxin before use

The concentration or capacity of the toxin may vary depending on the degree of wrinkles, muscle mass, and experience of the physician. However, the fundamental thing is to use a high concentration of toxin in the cases when the muscles are thick and small and the toxin should not be migrated, and to use a low concentration of toxin when the injection area is relatively wide and the toxin migration does not cause a serious complication [29].

For reference, when injecting the high concentration, the author mixes 500 U of Dysport with 2.5 cc of injectable normal saline and 50 μg/ml of epinephrine (expecting vasoconstriction) and injects the anomalous concentration (0.1 cc = 2 U). When lower concentration is required, the author mixes the same amounts of toxin and injectable normal saline and makes the concentration of 0.01 cc to be 1 U.

#### Applicability in maxillofacial area

Applicability of this toxin in the maxillofacial area or dental area shows the excellent effect in removing wrinkles in the forehead, eye rim, middle of the forehead, nose bridge, etc., is being effectively used in the treatment of square jaws with masseter muscle hypertrophy and can achieve satisfactory effect in the patients who have prominent mandibular angle but do not want surgery [30].

Moreover, injecting toxin to a normal or hyperactive nerve can maintain the symmetry of the lip movement in the cases of marginal mandibular branch paralysis or facial nerve hyperactivity, and injecting toxin to an enlarged area can restore the symmetry in the case of facial asymmetry. In addition, the recent application of the toxin to temporomandibular disorder (TMD) that does not respond to the traditional treatment and is accompanied with teeth-grinding myotonic pain has yielded satisfactory results [31]. It is thought that its applicability in the dentistry will increase further.

If your purpose of use is to reduce the activity of muscles and relieve pain occurring in the temporomandibular joint and head and neck region, you can use botulinum toxin according to the following classification:

- *Jaw-closing muscles*. Temporalis, masseter, medial pterygoid muscle
- *Jaw-opening muscles*. Suprahyoid muscles, lateral pterygoid muscle
- *Muscles prominent in cervical regions*. Sternocleidomastoid muscle, trapezius, paracervical musculature
- *Muscles prominent in cephalic and facial regions*. Frontalis, occipitalis, procerus, corrugator

Table 2 summarized the origin and insertion site of masticatory muscles and dose of botulinum toxin injection.

**Table 2:** Masticatory muscles and corresponding dose of botulinum toxin

Muscle	Origin	Insertion	Dose	Number of injection
Temporalis	Temporal fossa	Medial and anterior aspect of coronoid process of mandible	5~25 BU	5
Masseter	Anterior two thirds of zygomatic arch and zygomatic process of maxilla	Lateral surface of angle and lower ramus of mandible	25~50 BU^a^	5
Medial pterygoid	Deep head: medial side of lateral pterygoid plate and fossa between medial and lateral plates. Superficial head: tuberosity of maxilla and pyramidal process of palatine bone	Medial aspect of angle of mandible	5~25 BU	2~3
Lateral pterygoid	Upper head: infratemporal surface of sphenoid bone. Lower head: lateral surface of lateral pterygoid plate	Pterygoid fovea below condyloid process of mandible and TMJ meniscus	5~10 BU	1

^a^Dose may vary depending on the purpose of use. 25 BU is used for the reduction of masseter muscle activity

#### Complications

In terms of the side effects of botulinum toxin use, immunization can occur when this toxin is used too often. In order to prevent this, the minimum effective dose should be used, and, if possible, the injection interval should be set at more than 3 months, and the frequency of the booster injection should be reduced. Also, the complications, that are occasionally observed when the injected toxin migrates to the surrounding areas, include brow ptosis, blepharoptosis, lagopthalmia, ectropion, facial nerve paralysis, etc. In addition, in rare cases, injections at the jaw or the neck may cause dysphagia or worsen dry eye syndrome [29].

## Conclusion

Botulinum toxin is a relatively safe and simple substance for use and has a very wide scope of application in maxillofacial or dental area, such as facial wrinkles, square jaw, facial asymmetry due to facial nerve abnormality, teeth grinding, and TMD. Therefore, this paper studied pharmacological mechanism, use instruction, usage dose, applicability in the maxillofacial area, complications, and others and expects to see lots of use in maxillofacial surgery and dentistry.

## Data Availability

Not applicable (data sharing not applicable to this article as no datasets were generated or analyzed during the current study).

## Abbreviations

BTX: Botulinum toxin
BU: Botox unit
DU: Dysport unit
LD: Lethal dose
TMD: Temporomandibular disorder
